# Evaluation of novel disposable bioreactors on pandemic influenza virus production

**DOI:** 10.1371/journal.pone.0220803

**Published:** 2019-08-12

**Authors:** Chia-Chun Lai, Tsai-Chuan Weng, Yu-Fen Tseng, Jen-Ron Chiang, Min-Shi Lee, Alan Yung-Chih Hu

**Affiliations:** 1 National Institute of Infectious Diseases and Vaccinology, National Health Research Institutes, Zhunan, Taiwan; 2 College of Life Science Biology, National Tsing Hua University, Hsinchu, Taiwan; 3 Vaccine Center, Centers for Disease Control, Taipei, Taiwan; University of Iowa, UNITED STATES

## Abstract

Since 1997, the highly pathogenic influenza H5N1 virus has spread from Hong Kong. According to the WHO bulletin report, the H5N1 virus is a zoonotic disease threat that has infected more than 850 humans, causing over 450 deaths. In addition, an outbreak of another new and highly pathogenic influenza virus (H7N9) occurred in 2013 in China. These highly pathogenic influenza viruses could potentially cause a worldwide pandemic. it is crucial to develop a rapid production platform to meet this surge demand against any possible influenza pandemic. A potential solution for this problem is the use of cell-based bioreactors for rapid vaccine production. These novel bioreactors, used for cell-based vaccine production, possess various advantages. For example, they enable a short production time, allow for the handling highly pathogenic influenza in closed environments, and can be easily scaled up. In this study, two novel disposable cell-based bioreactors, BelloCell and TideCell, were used to produce H5N1 clade II and H7N9 candidate vaccine viruses (CVVs). Madin-Darby canine kidney (MDCK) cells were used for the production of these influenza CVVs. A novel bench-scale bioreactor named BelloCell bioreactor was used in the study. All culturing conditions were tested and scaled to 10 L industrial-scale bioreactor known as TideCell002. The performances of between BelloCell and TideCell were similar in cell growth, the average MDCK cell doubling time was slightly decreased to 25 hours. The systems yielded approximately 39.2 and 18.0 μg/ml of HA protein with the 10-liter TideCell002 from the H5N1 clade II and H7N9 CVVs, respectively. The results of this study not only highlight the overall effectiveness of these bioreactors but also illustrate the potential of maintaining the same outcome when scaled up to industrial production, which has many implications for faster vaccine production. Although additional studies are required for process optimization, the results of this study are promising and show that oscillating bioreactors may be a suitable platform for pandemic influenza virus production.

## Introduction

Since the avian influenza H5N1 outbreak of 2003, the H5N1 virus has caused over 450 deaths [1]. In addition, the avian influenza H7N9 virus has caused outbreak in China. The flu vaccine for unmatched strains of the virus is not expected to be cross-protective confirmed by data relating to the H5N1 pandemic strain. Many animal and clinical-trial studies have shown that the 2004 H5N1 influenza vaccine virus strain, which belongs to the first H5N1 genotype (clade I), does not provide cross-protection for the most recently isolated H5N1 virus from the Chinese mainland and Hong Kong, which belongs to the second H5N1 virus genotype (clade II) [2, 3]. To prevent influenza outbreaks from spreading, the most effective public health measure is vaccination [4]. Currently, influenza vaccine production heavily relies on traditional embryonated egg technology [5]. This process requires long and logistic planning that would severely delay the vaccine production to meet the surge demand in the event of a pandemic. Cell-based technology is considered as an alternative platform for influenza vaccine production, and it has piqued the interest of many in recent years [6, 7]. The common cell lines used for cell-based influenza vaccine production are MDCK (derived from Madin-Darby canine kidney) and Vero (derived from African green monkey kidney) cells, which are anchorage-dependent cells [8, 9]. For influenza vaccine production, it is crucial to choose a system, which is simple and robust, can produce high viral titers from a wide variety of influenza virus strains [10]. A number of cell culture systems were already used for their large-scale vaccine production potential, such as roller bottles and cell factories. These systems were originally designed for adherent cells; however, large-scale production with these systems is challenge to increase surface to volume ratio for cell proliferation. A solution to overcome this problem would be to use a microcarrier cell-lift bioreactor (New Brunswick Scientific, USA), by providing good mixing of the oxygen supply and a high concentration of microcarrier for more surface area. Other traditional bioreactors such as hollow-fiber bioreactors [11], the Celligen Plus bioreactor, [12] or bioreactors supplemented with microcarriers were already used for large-scale production [13]. However, all of these bioreactors involve complicated operations and are labor intensive. Since single-use (disposable) bioreactors were introduced, the traditional stainless-steel bioreactors slowly became obsolete in small-scale biotechnology and contract manufacturing companies [14]. Single-use bioreactors offer lower capital cost, easier operations, faster turn-around times, and fewer requirements for cleaning validation. Two novel bioreactors, BelloCell (bench-top scale) and TideCell002 (industrial scale), have recently been developed by Cesco Bioengineering, Taiwan. The BelloCell bioreactors have been successfully used to cultivate mammalian cells for the production of HDV-like particles [15], Japanese encephalitis virus [16], and insect cells for baculovirus [17]. In these studies, the bioreactors have consistently shown various beneficial characteristics: (1) Improved efficiency because they are pre-sterile and ready-to-use, (2) low shear stress because they move the liquid gently without the generation of gas bubbles, (3) large surface area to achieve a high-density growth of cells, and (4) the ability to collect whole cells or cell components [15, 18, 19]. At present, the use of these single-use bioreactors for influenza vaccine production has not been reported. In this study, we assessed the feasibility of commercially available disposable bioreactor systems for vaccine development. We used the H5N1 clade II and H7N9 candidate vaccine viruses cultured in both MDCK and Vero cells. The results of this study should illustrate the effectiveness of the disposable bioreactors and highlight the potential of their scalability for industrial use.

## Materials and methods

### Cells lines, virus strains and medium

Vero cells (CCL-81), a continuous African Green Monkey kidney cell line, and MDCK cells (ATCC CCL-34), a continuous Madin Darby Canine Kidney cell line, were purchased from the Food Industry Research and Development Institute, Hsinchu, Taiwan. For the Vero and MDCK cells growth and virus replication stages, VP-SFM and OptiPRO serum-free medium (Invitrogen, California, USA) supplemented with 4 mM L-glutamine was used, respectively. The MDCK cells were used at passage number between 63 and 69. The Vero cells were used for all experiments at passage number of 131 to 150. Two H5N1 candidate vaccine viruses, an RG6 strain derived from A/Anhui/01/2005(clade 2.3.4) and an RG30 strain derived from A/Hubei/1/2010 (clade 2.3.2.1) were obtained from the US CDC and were re-adapted for use in MDCK cells. The H7N9 strain (RG268) derived from A/Anhui/01/2013 was obtained from the NIBSC, UK and adapted for use in MDCK cells. The re-adapted strains were obtained by choosing better replication colonies in the plaque assay. The antigenicity of the chosen colonies was confirmed by the HI assay. Generally, the process needs to be repeated 3 to 10 times before obtaining the high growth CVVs in the MDCK cell culture. In this study, H5N1 adapted to MDCK cell for passage 10 times, and H7N9 adapted to MDCK cell for passage 5 times.

### Spinner flask cultures

The Cytodex 1 microcarriers (GE Healthcare, USA) used for cell immobilization were made of cross-linked dextran matrix with a specific surface area of approximately 4400 cm^2^/g. The culturing method was described in the previous study [10]. The microcarriers were hydrated, autoclaved and preconditioned according to the manufacturer’s instructions before use. The cultures were performed in 1-L spinner flasks with a working volume of 400 mL, and each spinner flask contained 5 g/L of Cytodex 1 microcarriers. Each flask was initially inoculated with approximately 2 x 10^5^ cells/mL. During cell growth, the pH, glucose, and glutamine concentrations were monitored daily, and the medium exchange rate was adjusted daily to maintain the glucose concentration at approximately 1 g/L. In addition, samples were taken daily to determine cell density. The concentration of cells for viral infection was approximately 2 x 10^6^ cell/mL on day 4 after seeding. The cells were spun down, washed three times with 100 mL of PBS, and then approximately 75% of the culture medium (300 mL) was exchanged with fresh medium. In addition, the cultures were supplemented with 2 μg/mL of TPCK-treated trypsin (Invitrogen, USA) for viral growth. The cells were infected with candidate influenza H5N1 vaccine viruses with a multiplicity of infection (MOI) of 10^−4^. During the viral production, the culturing conditions were maintained at pH 7.2 and 34°C in a incubator with 5% CO_2_. All of the virus-containing broth, without microcarriers, was harvested when the viral titer reached the peaked on 3 days post-infection (dpi). Each batch run was repeated twice.

### Bench-top scale BelloCell bioreactor run

**Fig 1:**
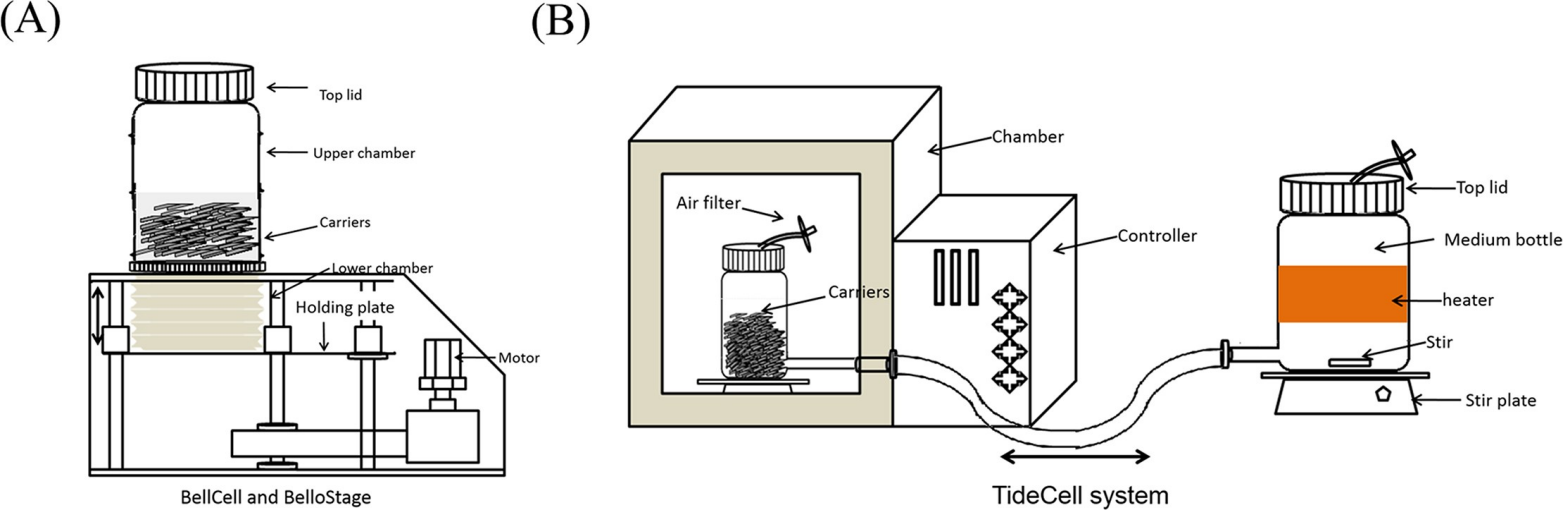
Schematic diagram of the BelloCell and TideCell systems. The BelloCell (A) and TideCell (B) systems consist of the control stage and the culturing bottle. The cultured medium level rises and descends periodically by the control stage motor. During the aeration, cells attached to the porous matrices are exposed to air for gas exchange. In the submerging phase, cells directly contact with liquid for fresh medium and waste exchange.

The BelloCell bioreactor system has been used for the development of Vero cell-based Japanese encephalitis vaccines since the early 2000s. The BioNOC II (Cesco Bioengineering Co., Taiwan) carriers were used for cell attachment and were made out of 100% polyethyleneterephthalate (PET) non-woven fabric strips (width ~5 mm, length ~10 mm), with a specific surface area of approximately 2400 cm^2^/g. The fabric was surface-treated to make it hydrophilic and biocompatible. Each bottle of BelloCell-500 was pre-packed with ~865 BioNOC II carriers (approximately 5.5 g/pack, and ~100 cm^3^ bed volume). BioNOC II carriers were placed in a BelloCell-500 bottle and pre-sterilized by γ-irradiation. The BelloCell-500 bottle contains two parts, the upper chamber, used for cell immobilization, and the lower chamber, used to hold medium. There are two types of BelloCell-500 bioreactors, the BelloCell-500P and the BelloCell-500A. The BelloCell-500P bottle is equipped with an upper tubing (inlet) and a lower tubing (outlet) for perfusion culture use. Fresh medium was gradually introduced into the lower part of BelloCell-500P, and the medium was circulated by a peristaltic pump in the BelloFeeder. The BelloCell-500P system used a total of 2200 mL of medium. The BelloCell-500A bioreactor is designed for batch culture operation with working medium volume of 500 mL was used. Instead of the BelloFeeder, the movement and displacement of the lower chamber was controlled by a BelloStage console (Fig 1A). The concentration of inoculated cells was 2 x 10^5^ cells/mL. During cell growth, the pH and glucose concentration were monitored once a day, and when glucose concentration dropped below 1 mM/L, the culture was replenished with fresh medium. The pH value was maintained at 7.0 by adjusting with 5% CO_2_ in the BelloCell-500 and/or adding NaHCO_3_ in the medium, with samples taken daily to determine cell density. When the cell concentration reached approximately 2 x 10^6^ cells/mL, the medium was exchanged for fresh medium supplemented with 2 μg/mL of TPCK-treated trypsin. The cells were infected with influenza H5N1 vaccine viruses with an MOI of 10^−4^. During the virus harvest period, the medium was not changed, and the entire harvest was collected when the viral titer peaked. Each batch run was repeated twice.

### Industrial-scale TideCell002 bioreactor run

The TideCell002 bioreactor operates under the same principle as the BelloCell bottles. Each TideCell002 is pre-packed with approximately 55 g of BioNOC II carrier chips and pre-sterilized by γ-irradiation. The culture medium is periodically transferred from the external medium bottle into the carrier matrix vessel and is then withdrawn from the vessel using applied air pressure to export the matrices with the carriers. Thus, cells entrapped inside the matrices are able to receive sufficient nutrients and oxygen, while metabolic wastes, such as lactate and carbon dioxide, can be efficiently carried away in a gentle manner (Fig 1B). The culture medium was exchanged by the external mixing bottle, homogenized by different agitation methods, and circulated between the matrix vessel in the incubator and the mixing bag. This is accomplished by the switch between negative and positive air pressure, which submerges the matrices where the cells attach. In this study, the working liquid volume of the bioreactor was 10 L.

### Calculation of cell numbers and distribution on BioNOC II carriers

To count cells on the BioNOC II carriers, a nucleus-staining crystal violet dye (CVD) method was used. To count cells on BelloCell-500A, six carrier chips were sampled from different locations (top, middle, and bottom) of the bottle and placed in three 1.5 mL Eppendorf tubes, with two carrier strips in each vial. After adding 1.0 mL of CVD reagent, each vial was shaken and incubated at 37°C for 20 min, which was repeated three times to ensure that the cell membranes ruptured and that the nuclei were released from the strips. To analyze MDCK cell distribution on BioNOC II carriers during the culturing period, the four bottles of BelloCell-500 bioreactors were seeded at the same cell concentration. Each BioNOC II carrier (approximately 865 carriers) of each bottle was counted daily and monitored by the CVD staining method in a 96-well plate from day 1 to 4. A hemocytometer was used to perform the nucleus count, allowing the cell numbers on each BioNOC II carrier to be indirectly counted.

### Analysis of cell metabolites in the BelloCell bioreactor

The pH value and the concentration of glucose, lactate, glutamine, glutamate and ammonia in the culture supernatant was measured offline using a NOVA Bioprofile 400 biochemical analyzer (Nova Biomedical Corporation, USA). The glucose uptake rate (GUR) was monitored on the growth curve of MDCK cells and was calculated every day.

### Virological assays

Hemagglutinin (HA) titration was conducted in 96-well microplates using turkey red blood cells (RBC) following standard protocols [20]. Virus infectious titers were measured using the 50% tissue culture infectious doses (TCID_50_) assay based on the cytopathic effect (CPE) in MDCK cells. A positive control with a pre-specified acceptable range was included for conducting HA and TCID_50_ assays [21].

## Results

### Cell growth in the BelloCell bioreactor

**Fig 2:**
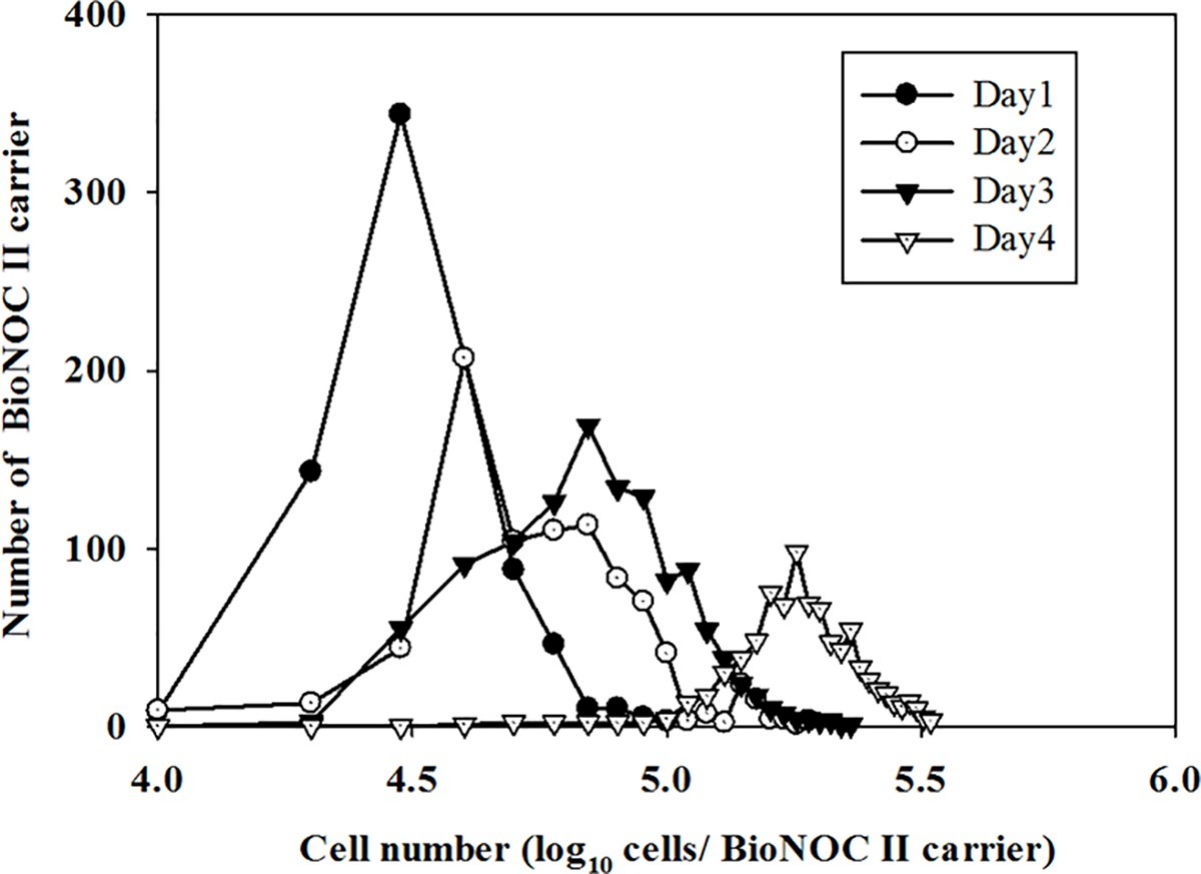
Time dependent analysis of MDCK cells distribution on BelloCell. The MDCK cells were inoculated with a cell density of 2x10^5^ cells/mL in Bellocell. The cells that counting the number of cells per day. The cell numbers were counted in each fabric on day 1(●), 2(○),3(▲) and 4(△). The total number of fabric are about 860 in the Bellocell.

In the cell-based platform for growth of influenza viruses, the common cell lines used are MDCK and Vero. We first wanted to evaluate to the growth of these two cell lines in the BelloCell bioreactor. The MDCK cells were grown in a 500 ml BelloCell-500A bioreactor with the OptiPRO-SFM, with an initial cell density 1 x10^5^ cells/ml. The density of the harvested cells reached 2.62 x10^6^ cells/ml on day 5. Vero cells were cultured in the VP-SFM using the BelloCell-500A, with an initial cell density 2 x10^5^ cells/ml. The density of the harvested cells reached 1.54 x10^6^ cells/ml on day 5. For the Vero and MDCK cells, the number of cells increased by 7.7- and 26.2-fold, respectively. This result showed that the MDCK cells can reach a higher cell density than Vero cells on the BelloCell-500A systems (Table 1). To confirm the ability of the MDCK cells to attach to the matrix and assess their degree of distribution, the cellular distribution of the matrices was analyzed in the BelloCell-500A. The MDCK cells were inoculated into the four bottles of the BelloCell-500A bioreactors, with an initial inoculum of 1 x10^8^ cells for each BioNOC II carrier (approximately 865 carriers) for each BelloCell-500A bottle, with cells counted and monitored daily. The cell number analysis showed that the total number of cells increase to 3.10x10^8^, 5.58 x10^8^, 9.34 x10^8^ and 1.62 x10^9^ cells on days 1, 2, 3 and 4, respectively. The cultured cell distribution had a coefficient of variation (% CV) of 2.54, 1.61, 1.36, and 1.03 on days 1, 2, 3, and 4, respectively. The time course data showed that the cellular distribution tended to become more uniform with increasing culture time (Fig 2). These results indicate that the MDCK cells showed good growth ability in the BelloCell bioreactor. Thus, subsequent experiments used the MDCK cells as a host for influenza virus production.

**Table 1:** Performance of MDCK and Vero cell growth in a BelloCell-500A bioreactor.

Total surface area (cm^2^)	15,600	
Working volume (mL)	500	
Cell line	MDCK cell	Vero cell
Seeding cell density (cells/mL)	2.0x10^5^	2.0x10^5^
Total usage medium (mL)	2,000	1,500
Cell doubling time (hour)^1^	25.87	44.39
Harvest cell density (cells/mL)	2.62x10^6^	1.54x10^6^
Fold increase of cell growth	13.1	7.7

1. Calculate the cell doubling time with the following formula: Cell doubling time = during time (T) x ln2/ln (Xe/Xb); T is the incubation time in any units.; Xb is the cell number at the beginning of the incubation time.; Xe is the cell number at the end of the incubation time.

### Metabolic kinetics of the Vero and MDCK cells in the BelloCell-500A

**Fig 3:**
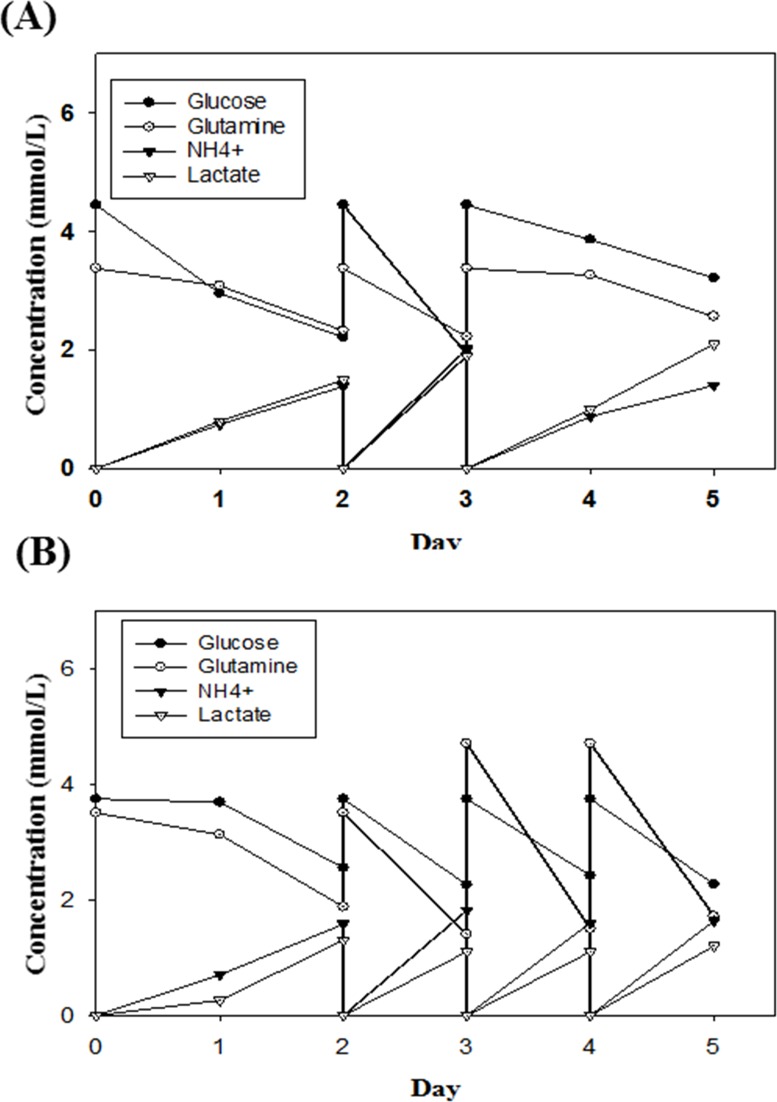
**Metabolic profile of MDCK (A) and Vero (B) cells grown in the BelloCell-500A bioreactors.** The MDCK (A) and Vero cells (B) cells were cultured in 500 mL BelloCell. The MDCK and Vero cells were inoculated with a cell density of 2x10^5^ cells/mL. The Metabolic profile (○): Glucose, (●): Glutamine, (▲): NH_4_^+^, (△): Lactate. were monitored during the culture period.

To investigate culture conditions for the two cell lines in the BelloCell-500A bioreactor, the metabolic kinetics of MDCK cells using OptiPRO™-SFM were first evaluated. The initial glucose and glutamine concentrations were approximately 3.5 and 3.8 mmol/L, respectively, with the glucose concentration maintained at 1.0 mmol/L throughout the experiment. The medium was exchanged on days 2, 3 and 4 to avoid a shortage of essential nutrients and the accumulation of toxic metabolites (Fig 3A). During culturing of the MDCK cells, a total of 2 L of fresh OptiPRO-SFM was used to maintain sufficient nutrients and waste reduction in the culture medium. The GUR peaked after the second day and rapidly declined thereafter. The metabolic kinetics of Vero cells in VP-SFM were also evaluated in the BelloCell-500A, with initial glucose and glutamine concentrations maintained at 3.5 and 4.5 mmol/L, respectively. The prevention of nutrient depletion and waste accumulation in the Vero cells was followed by a similar protocol as the MDCK cell cultures, with the medium exchanged on days 2 and 3 (Fig 3B). During growth of the Vero cells, old medium was replenished with 1.5 L of fresh medium. The GUR increased over time. These data showed that the metabolic kinetics of the MDCK cells was more exuberant than that of Vero cells under the same culture conditions, and this phenomenon was correlated with the observation of fold increase in cell growth.

### Evaluation of the production of different influenza vaccine strains using the BelloCell-500A bioreactor

In cell culture experiments, the MDCK cells were suitable for virus replication using the Beollcell-500A. To determine the production of influenza vaccine strains in the Bellocell-500A, different pandemic influenza vaccine strains were used in this study and the result were compared with a spinner-flask production system. The MDCK cells were grown in a 500 ml BelloCell-500A system with OptiPRO-SFM. The initial cell density was 2x10^5^ cells/mL, and the average cell density reached 2.62x10^6^ cells/mL on day 4. MDCK cells were also cultured in a 400 mL spinner flask containing a 5 g/L Cytodex 1 microcarrier, and the average cell density reached 1.98x10^6^ cells/mL on day 4. The MDCK cells in the BelloCell-500A were infected with influenza vaccine viruses using a very low MOI of 10^−4^. For virus production, the temperature was adjusted to 34°C, and the total cytopathic effect (CPE) was observed in the MDCK cells on day 3. The MDCK cells were infected with influenza H5N1 clade II vaccine strains (RG6 and RG30) and influenza H7N9 vaccine strains (RG268) from cell-adapted virus candidate strains. The results showed that the HA titer of RG6, RG30, RG268 was 1024, 64 and 512, respectively, and the TCID_50_ titer was 3.98x10^9^, 6.00x10^6^ and 1.00 x 10^7^ virion/ml, respectively. The ability of the 400-ml spinner flask containing 5 g/L Cytodex 1 microcarriers system and the BelloCell-500A to produce viruses was compared. The data revealed that TCID_50_ titer in the spinner-flask system with the three tested strains was higher than that in the BelloCell-500A, but the data did not correlate well to the HA titers. The results showed that the HA titers were similar between the BelloCell-500A and the spinner flask culture (Table 2). The results of this analysis showed that the BelloCell-500A bioreactor had a good ability to produce pandemic influenza virus vaccine strains.

**Table 2:** Cell-specific productivity comparison of MDCK cell grown on BelloCell 500A or spinner flask bioreactors.

	BelloCell 500A				Spinner-flask			
			Cell-specific productivities^3^				Cell-specific productivities	
Virus strain^1^	HA^2^ units/100μL	TCID_50_ virions/mL	HA units/cell	TCID_50_ virions/cell	HA^2^ units/100μL	TCID_50_ virions/mL	HA virions/cell	TCID_50_ virions/cell
H5N1 (RG6)	1024	3.98 x 10^9^	7817	1519	1024	1.00 x 10^10^	10343	5050
H5N1 (RG30)	64	6.00 x 10^6^	489	2	64	3.98 x 10^7^	646	20
H7N9 (RG268)	512	1.00 x 10^7^	3908.	4	256	5.62 x 10^7^	2589	28

1. The virus strains (RG6, RG30 and RG268) were already adapted in MDCK cells.

2. Maximum HA titer expressed as HA units/100μL.

3. Cell-specific productivities were calculated from HA or TCID_50_ value and cell concentration at time of infection: HA was converted to virions/mL, assuming the binding of one virus particle per red blood cell (RBC) at a given RBC concentration of 2.0x10^7^cells/ml; the other calculation method was calculated from TCID_50_ titer as live virus particle number and cell concentration at time of infection.

### Evaluation of the influenza H5N1 clade II vaccine strain on perfusion run

The BelloCell bioreactor has the ability to provide a large surface area to achieve high-density cell growth. However, the high-density cell growth causes the initial medium to be insufficient. The BelloCell-500A uses medium replacement to solve the nutrient deficiencies during cell culturing. Another system, the BelloCell-500P, has a total medium volume of up to 2200 mL via the perfusion system. The perfusion system provides another way to maintain sufficient nutrients and waste reduction in the culture system. To evaluate the productivity of both systems, the influenza H5N1 clade II (RG6) vaccine strain was used as an example to compare the different BelloCell systems. The MDCK cells were grown in OptiPRO-SFM using the 500-ml BelloCell-500A system and the BelloCell-500P system. The initial cell density was 2 x10^5^ cells/mL, and after four days, the cell density reached 2.6 x10^6^ cells/mL and 7.65 x10^5^ cells/mL for the BelloCell-500A and BelloCell-500P systems, respectively. The data showed that the cell density in the BelloCell-500P system was lower than that in the BelloCell-500A system. However, the total number of cells was higher in the BelloCell-500P system than in the BelloCell-500A (Table 3). The MDCK cells in the BelloCell-500A and BelloCell-500P systems were infected with influenza H5N1 clade II (RG6) virus, and the total CPE was observed on day 3. The virus titer was analyzed by HA and TCID50 assays. The virus titer data indicated that the virus productivity in the BelloCell-500P system was higher than that in the BelloCell-500A under the same conditions (Table 3). The productivity results showed that perfusion systems are more suitable for influenza vaccine production.

**Table 3:** Evaluation of influenza H5N1 clade II virus (RG6M13C4) production in different BelloCell-500 systems.

	Volume (mL)	HA titer^1^ (units/100μL)	TCID_50_ (virions/mL)	Cell destiny (cells/mL)	Cell-specific productivities^2^	
					HA virions/cell	TCID_50_ virions/cell
T75 flask	20	128	2.93 x10^7^	5.00 x10^5^	5120	59
BelloCell-500**A**	500	512	3.16 x10^7^	2.60 x10^6^	3938	12
BelloCell-500**P**	2200	512	2.50 x10^7^	7.65 x10^5^	13386	33

1. Maximum HA titer expressed as HA units/100μL.

2. Cell-specific productivities were calculated from HA or TCID_50_ value and cell concentration at time of infection: HA was converted to virions/mL, assuming the binding of one virus particle per red blood cell (RBC) at a given RBC concentration of 2.0x10^7^cells/ml; the other calculation method was calculated from TCID_50_ titer as live virus particle number and cell concentration at time of infection.

### Scaling-up study from the BelloCell-500P to the TideCell002 bioreactor

A good ability to produce the pandemic influenza vaccine strains was obtained in the BelloCell-500P bioreactor. To evaluate the linear capability of a larger production scale, the TideCell002 bioreactor was assessed for virus production. The TideCell002 was able to scale-up to over a 10-L production capacity. The MDCK cells were grown in a TideCell002 (with 55 g of BioNOC II carriers) bioreactor system with OptiPRO-SFM. The initial cell density was 2 x10^5^ cells/mL, and the average doubling time of MDCK cells was 27.57 hours. The cell density reached 2.62 x10^6^ cells/mL on day 4. The metabolic kinetics of MDCK cells in OptiPRO-SFM were also evaluated in the TideCell002. The initial glucose concentration was approximately 3.5 mmol/L and was maintained at 1.0 mmol/L. The lactate concentration was also monitored to ensure that the lactate concentration did not go over 1.5 mmol/L during the experiment. Ten liters of medium was replaced on day 3 to avoid a shortage of essential nutrients and avoid the accumulation of toxic metabolites. Twenty liters of OptiPRO-SFM was used in total for MDCK cells. The GUR of MDCK cells was assessed and ranged from 120 to 370 mg/h in the TideCell002 system. The maximal GUR was calculated to be 370 mg/h at 96 hours and remained steady thereafter. According to our pilot study, the conditions for the infection of MDCK cells in the TideCell002 bioreactor were similar to the BelloCell, except the total cytopathic effect (CPE) was observed on day 4. The MDCK cells were infected with the influenza H5N1 clade II (RG6) and influenza H7N9 (RG268) virus candidate strains. The results showed that the HA and TCID50 of RG6M13C4 were 512 and 1 x10^8^ virion/mL, respectively, while the HA and TCID50 of RG268M5 were 512 and 3.16 x10^7^ virion/mL, respectively (Table 4). These results demonstrated that high-yield MDCK cell-based influenza H5N1 and H7N9 vaccine production is possible using the TideCell002 bioreactor system and has a good linear amplification scalability.

**Table 4:** Scaling-up evaluation of pandemic influenza virus production in TideCell002 bioreactor.

Virus strain	Volume (mL)	HA titer^1^ (units/100μL)	TCID_50_ (virions/mL)	Cell destiny (cells/mL)	Cell-specific productivities^2^	
					HA virions/cells	TCID_50_ virions/cells
H7N9	10,000	512	1.00 x10^8^	1.98x10^6^	5120	51
H5N1^3^	10,000	512	3.16 x10^7^	1.98 x10^6^	5120	16

1. Maximum HA titer expressed as HA units/100μL.

2. Cell-specific productivities were calculated from HA or TCID_50_ value and cell concentration at time of infection: HA was converted to virions/mL, assuming the binding of one virus particle per red blood cell (RBC) at a given RBC concentration of 2.0x10^7^cells/ml; the other calculation method was calculated from TCID_50_ titer as live virus particle number and cell concentration at time of infection.

## Discussion

Cell culture processes have played an increasingly important role in the development of influenza vaccines. MDCK cells have been used in the formulation of the vaccine Flucelvax Quadrivalent (Seqirus, Inc), which is approved for use in Europe and the USA [22]. However, the development of an efficient cell culture process is still needed. It is important that cell-based influenza vaccine manufacturing processes use a highly efficient bioreactor that can produce high cell densities, high virus yields and high HA titers from a wide variety of influenza virus strains. In a study by Hu *et al*., the BelloCell was shown to have a good gas exchange ability and high cell density culturing [23]. In this study, we confirmed the ability of the BelloCell system to culture mammalian cells at high cell densities. The BioNOC II carriers in the BelloCell allow the cells to attach and proliferate (Fig 3). In recent years, commercially available cell-based influenza vaccines are commonly produced from either Vero or MDCK cells. In this study, we showed that MDCK cells achieved a higher cell density than Vero cells in the BelloCell system, suggesting that the MDCK cell line is more suitable for use with the BelloCell system (Table 1). In addition, recent studies have shown that MDCK cells are the most suitable for propagating influenza viruses, as the cells not only enable the growth of different influenza virus strains but also produce good virus yields with relatively high HA titers [24–26].

The cell culturing and virus replication conditions for influenza vaccine production were established using the method described in the microcarrier process by Hu *et al*., 2011. In this study, the results show that the HA titers were similar between the BelloCell and microcarrier processes (Table 2). We confirmed that the BelloCell system has the ability to produce high yields of influenza viruses and showed that the production system has a high potential for cell production capacity. The observed level of cell production is similar to that observed in previous studies and has been successfully applied to the production of different viruses [15, 16, 27]. In addition, about spinner-flask has a consistently higher TCID_50_ titer than that in the BelloCell-500A.This is an interesting phenomenon. The TCID_50_ titer results indicated that the total number of live virus, not total the effective antigen. The HA titer could be explaining to the total number of effective antigen in this study. In the virus production curve data showed the TCID_50_ titer was decreasing from 48 hours. However, the HA titer was still remaining high till 72 hrs (S1 Fig). These data maybe can explain that the spinner-flask have a more live virus, but the total number of the virus was similar to BelloCell-500A.

In previous reports [10, 28], MDCK and Vero cells could produce influenza viruses using SFM in spinner flasks. In this study, the same cells were used for the production of H5N1 influenza clade II and H7N9 vaccine strains using SFM in the BelloCell systems, as well as in spinner flasks. The cell density reached in the BelloCell system was higher than that of the spinner flasks; however, the virus titers were similar in both systems. This suggests that cell density does not correlate with virus titer well. This may be due to the “cell density effect”, which was first reported by Wood *et al*., 1982. The cell culture was observed to be "nutrient-limited", and this nutrient limitation caused total cell yield and health to be compromised. The nutrient limitation and unknown inhibitory factors may have contributed to the decrease in virus productivity in the culture medium [29]. Another study encountered a similar problem when dealing with adenovirus [30] and influenza virus [31, 32]. In this study, the ability of the BelloCell-500P (2200-mL) and the BelloCell-500A (500-mL) to produce influenza virus was compared. It was observed that increasing the volume of the culture medium resulted in a three-fold increase of influenza virus production per unit cell in the BelloCell-500P (Table 3). Thus, we confirmed that the cell density effect played a key role in BelloCell experiments, and nutrient limitation may be one of the important factors affecting the yield. In addition, the volume of the culture medium also reflection on the price. It could be compared by the usage of medium and HA titer produced. The data showed that Beollcell-500p bioreactors maybe have cheaper than flask system and Beollcell-500A in well-control condition. This information was shown in Table 5.

**Table 5:** Productivity comparison of the virus production in different systems.

	BelloCell 500P	BelloCell 500A	Spinner flask
Medium name	OptiPro-SFM^3^		
Working volume (L)	2.2	0.5	0.4
HA titer (HA units/100μl)^1^	512	512	512
Medium usage volume (L)	4.4	2.0	2.0
Specific productivity (HA unit/L)^2^	256.0	128.0	102.4

1. HA titer as HA units/100μL (RBC concentration:2.0x10^7^cells/ml)

2. The specific productivity is based on the calculation of viral titer * working volume /medium usage.

3. The cost based on OptiPro-SFM only, about 100 US/L.

As described by *Merten et al*., *2015* [33], the difference in bioreactor system design has caused bench-scale process parameters did not correlated well to industrial-scale bioreactors. The shear force generated from the agitating blade and the uniformity of the cell adhesion are often an issue, resulting in a decrease in the production process. In this study, different influenza virus vaccine strains were cultured in the BelloCell and in spinner flasks with microcarrier. The TideCell002 was also used, and its entire process is amplified in volume by almost 20-fold compared to the BelloCell-500A system. Based on similar culture conditions, the HA titer did not differ significantly. In many previous reported, these virus production systems have been widely used to produce a vaccine[34]. However, since an influenza pandemic usually spreads quickly and is highly contagious, a single-use system with rapid vaccine-production capabilities seems to be preferable. The results of this study have highlighted the potential for rapid and cost-effective vaccine manufacturing using the TideCell002 system. TideCell002 can produce 10 to 20 L of vaccine per run. In addition, the TideCell002 system has shown a good linear scale-up, and is easy to switch to larger scale bioreactors (TideCell100, equivalent to 500 to 1000 L, industrial scale). This novel single-use oscillating bioreactor is able to produce large quantities of vaccine antigen at an industrial scale (up to 1000 L).

## Conclusions

The goal of this study was to evaluate cell-based influenza virus production platforms using novel, single-use bioreactors, particularly for newly emerging influenza viruses, such as H5N1 and H7N9. BelloCell, a new type of bioreactor, has high cell density and viral production capabilities. The BelloCell is easily scaled up to the size of the TideCell system using similar operational conditions. This novel bioreactor shown good performance for adhesion cells for influenza virus production. This study highlights the ability of disposable bioreactors to manufacture vaccines quickly and efficiently. TideCell bioreactor could be quick and easy producing large amounts of the virus is a major point of the study. In future studies, The TideCell002 also shown a good scalability performance from the BelloCell bioreactor. If the TideCell bioreactor can also scaled to 1000 L production scale (harvest volume). Rapid production of influenza virus would be possible to meet the surge demand against any possible pandemic outbreak. In addition, this novel escalatory bioreactor also applies to other vaccine processes such as rabies polio and EV71.

## Supporting information

S1 Fig The HA and viral titer during viral infection in the spinner flask and beollcell-500A. The MDCK cells were cultured in **beollcell-500A and spinner flask**. These cultured cells were infected by H5N1. During the infection period, the HA titer (■) and TCID_50_ (●) were monitored.
(TIF)
