# Supplementary figures and images for: Evaluation of novel disposable bioreactors on pandemic influenza virus production
Source: PLoS One. 2019 Aug 12;14(8):e0220803. doi: 10.1371/journal.pone.0220803 (PMC6690526; doi:10.1371/journal.pone.0220803)

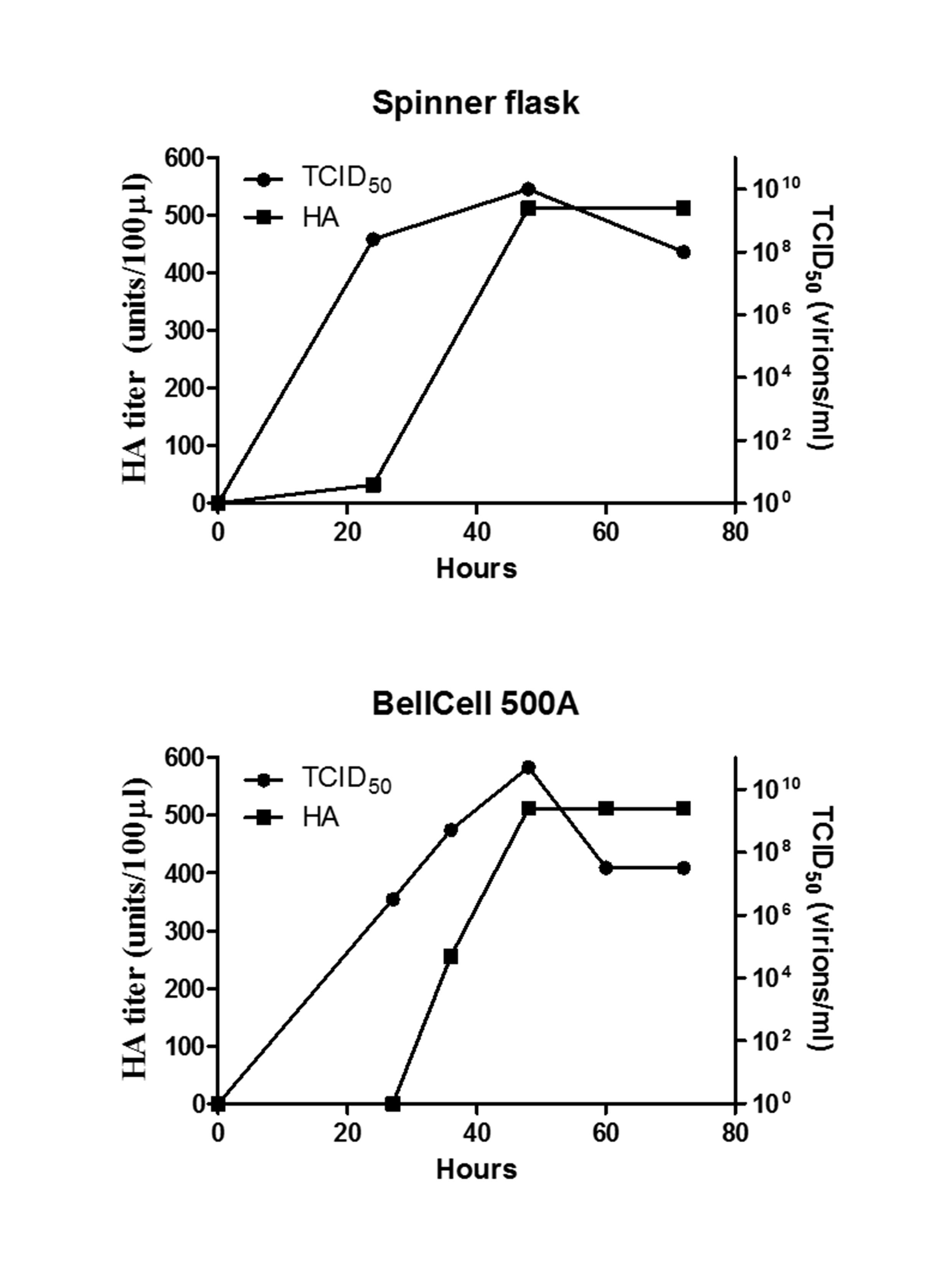

Supplement: S1 Fig — The MDCK cells were cultured in beollcell-500A and spinner flask. These cultured cells were infected by H5N1. During the infection period, the HA titer (■) and TCID50 (●) were monitored. (TIF) [file pone.0220803.s001.tif]
